# Risks of Aerosol Contamination in Dental Procedures during the Second Wave of COVID-19—Experience and Proposals of Innovative IPC in Dental Practice

**DOI:** 10.3390/ijerph17238954

**Published:** 2020-12-01

**Authors:** Maria Giovanna Gandolfi, Fausto Zamparini, Andrea Spinelli, Vittorio Sambri, Carlo Prati

**Affiliations:** 1 Laboratory of Green Biomaterials and Oral Pathology, School of Dentistry, DIBINEM, University of Bologna, 40126 Bologna, Italy; mgiovanna.gandolfi@unibo.it (M.G.G.); fausto.zamparini2@unibo.it (F.Z.); 2 Endodontic Clinical Section, School of Dentistry, DIBINEM, University of Bologna, 40125 Bologna, Italy; andrea.spinelli4@unibo.it; 3 Unit of Microbiology, DIMES, University of Bologna, 40125 Bologna, Italy; vittorio.sambri@unibo.it; 4 Unit of Microbiology, The Greater Romagna Hub Laboratory, 47822 Pievesestina, Italy

**Keywords:** SARS-CoV-2 infection, severe acute respiratory syndrome coronavirus 2, Coronavirus disease 2019 (Covid-19) diffusion, aerosol, droplets, dental offices, dental chair unit

## Abstract

Dental-care workers operate very close to the patient’s mouth and are at high risk of contamination by SARS-CoV-2. Droplets may be contaminated by patient’s saliva and exhaled breath particles. All asymptomatic patients should be considered as Coronavirus positive. All dental procedures must be revised after positive identification of SARS-Cov-2. Novel recommendations as the use of novel suction cannula designed for fast spray/saliva aspiration, use of Tyvek suits and innovative sprayhoods designed for dental-care worker protections are proposed to prevent virus transmission. New tailored operative and clinical procedures are being currently developed by university dental clinics and hospitals in attempt to reduce risk for dental workers and patients.

## 1. Introduction

A SARS-Cov-2 pandemic wave has spread across Europe and other world countries. It is expected that further pandemic recrudescence will occur in the next months and probably years.

Latest updates in Italy reveal an ever-growing number of positives, close to 40,000 cases per day during the month of November 2020. Therefore, it is very important that all patients treated in dental clinics must be pre-evaluated for triage to reduce and prevent the risk of contamination.

The large number of healthcare workers (HCWs) positive to SARS-CoV-2 in Italy during the current emergency was related to the work in high-risk areas—potentially infected environments—where aerosol-generating procedures are performed as in intensive care units, emergency rooms, departments of pulmonology and infectious diseases, and mostly in dental clinics. The face-to-face contact (within 0.5 m) between patients and dental care workers (DCWs) in the dental clinic represents a high-risk condition [1], that requires consideration and need for innovative countermeasures to limit viral contamination.

There is a need to study practical measures and predispose new guidelines for clinicians—specifically designed for each medical specialty—for the prevention of SARS-CoV-2 transmission, particularly in at-risk health dental care workers [2].

In the present commentary the authors want to perform a clinical picture of the dental procedures and focus on the several causes for high concern and to propose new devices to help to reduce the risk of contamination.

## 2. Situation during the Second Wave

The risk for HCWs and DCWs was extremely high during the first Covid-19 emergency. Both general practitioners and dentists faced a high number of deaths to Covid-19, revealing some of the most exposed medical professions during lock-down. The high number of affected DCWs closely mirrored the curve of new cases among Italian population, which confirmed that the exposure to SARS-CoV-2 also reflects a similar inauspicious outcome of doctors on duty [3].

To try to limit SARS-CoV-2 diffusion among dentists and patients, a number of clinical recommendations for a safe treatment during the Covid-19 emergency was reported [2,4]. This included a mandatory telephone triage to define the patients real need for emergency treatment and absence of recent exposition to Covid-19 positive patients, body temperature registration with a contactless thermometer to ascertain the absence of fever (<37.5 °C), hand disinfection, limit of patient number in the waiting room (one patient at a time) and removal of all potentially contaminated objects [2,4].

Unfortunately, the risk for virus transmission may still represent a tremendous hazard during the second waves of Coronavirus by the presence of a number of asymptomatic or paucisymptomatic patients still positive for SARS-CoV-2 [2].

An apparently healthy patient (positive for SARS-CoV-2) may potentially create the condition for a sequence of serious contamination of dental clinic personnel and other patients. This risk may be extremely high during SARS-Cov-2 s pandemic wave and will remain high until the vaccination of a large part of population. It is therefore important to design clinical solutions to prevent SARS-CoV-2 diffusion and to treat all patients and not only patient with dental emergency. These procedures must be maintained until the resolution of the pandemic emergency.

## 3. Routes of Infection in Dental Clinics

Nevertheless, the principal problem in dental clinics is constituted by the great number of aerosols and droplets generated by dental procedures and mixed with patient saliva and breath, which may contain a high number of pathogenic microorganisms, including bacteria (such as *Mycobacterium Tuberculosis*) [5,6] or viruses (such as influenza, measles or SARS-CoV-2) [7,8].

Dental chair unit (DCU) instruments, such as high-speed water-cooled hand pieces, ultrasonic instruments, low-speed polishing handpieces and dental air-water spray guns create a great number of water droplets and aerosols in front of the patient’s face [1,5,9].

The second important problem is connected with close (within 1 m) and prolonged contact of the operators with the patients. As example, an endodontic emergency for acute pulpitis may require at least 30–40 min but a normal session lasts more than one hour. Furthermore, the presence of 2–3 DCWs positioned close to the patient completes the description of the working area. High number of DCUs in close contact and not separated represents a great risk for virus diffusion, especially in presence of airflows that may diffuse droplets areas around dental equipment [10].

Therefore, after a single clinical session, all the operators are likely contaminated by a large number of aerosol particles (saliva, water, blood) produced by the DCU instruments on all protective surfaces as masks, goggles/face shields, gowns, glasses and also surfaces of equipment/furniture and floor. Conjunctiva and other part of the operator’s body may be exposed to the droplets and are at risk for contamination.

Other airborne particles—composed by dentin-enamel debris, dentin smear layer, fragments of composite/provisional cement/pastes—are usually produced into the mouth by the hand-pieces and remain suspended for a long time and diffused by air turbulences created by instruments and operator movements [1,5,9].

The presence of warm DCU lights may create convective forces that contribute to the diffusion of particles. Indeed, it is frequent to observe small droplets of saliva, water and blood into the external surface of light unit of DCU. New DCU LED lights may generate less heat than traditional halogen lights in relation to the convective forces that contributes to particles diffusion.

Contaminated hands are another important potential mode of virus transmission due to the frequent contact with bracket dental table, benchtops, DCU light handles etc. [1].

## 4. Droplets Movement around DC

Diffusion of droplets and aerosols around DCU has been previously reported [6]. Airborne transmission of infections refers to expelled particles that can remain suspended in air for long time (hours) and thus potentially expose operators at a great distance from the source of infection.

A recent study, which used adenosine triphosphate (ATP) bioluminescence as a biomarker of bacteria viability, found high levels of contamination on goggles and masks of dental operators and assistants [9].

Aerosolised viral particles may be potentially more dangerous than bacteria as they can remain airborne for longer periods of times, given the lower particle size, and the lower settling speed [8].

**Figure 1:**
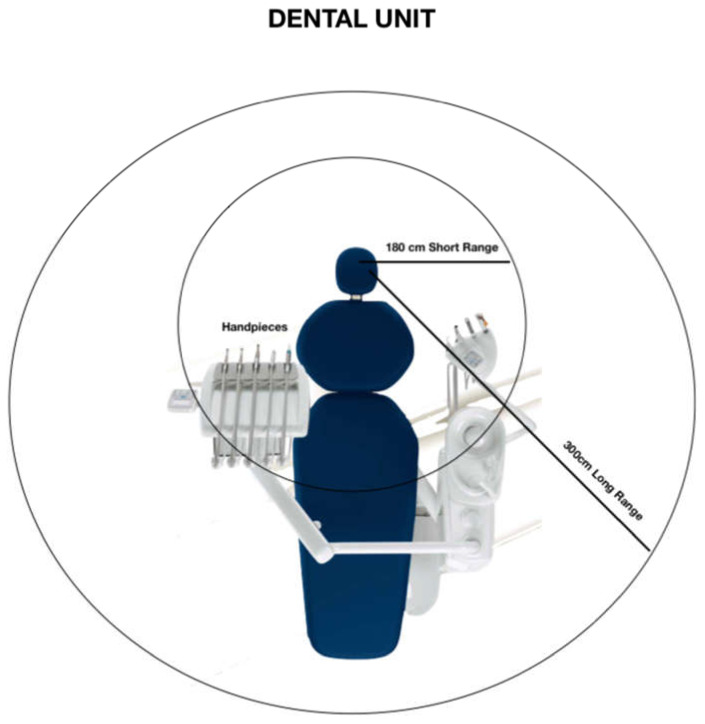
Areas of virus exposure around the dental unit.

The movements of operators just around the DCU create air turbulences with a traslocation of the aerosols and droplets from the area in front of the patient to somewhere in a range of meters (Figure 1).

The droplets have time enough to create an indoor airborne diffusion [9]. Normal speaking generates numerous small droplets (12–21 μm diameter prior desiccation), potentially containing encapsulated SARS-CoV-2 copies which remain airborne in a closed environment and able to reach patient and operator lower respiratory tract [9] (the use of ffp2 masks significantly reduce the risk of contamination).

Many variables are involved in the aerodynamics of particles. Air turbulence around the DCU like indoor temperature variations, air conditioning turbulence and door movements, may greatly influence the area and the surfaces where (infected) droplets may settle. In other words, aerosols and droplets may remain suspended in the air for hours inside the dental clinic and can be inhaled afterwards by DCWs and patients [11,12] and later may settle down on all surrounding surfaces and create many contaminated surfaces.

Droplets spread following a ballistic trajectory through the air with a deposition range of 3 feet. On the contrary, aerosol and droplet nuclei (dried particles) remain suspended in air and disperse over long distances following airflow streamlines and are inhaled and deposited in the human respiratory tract, from the glottis down to the alveolar space depending on their size and propulsive force [11,12]. The viral load entrapped in aerosols and droplet nuclei of about 1–5 micron in diameter, may be carried by air current and flow tides over considerable distance from the DCU [12].

Another critical variable that must be considered is the rate of particles desiccation. Particles begin desiccating immediately upon expulsion into the air, with a rate mainly depending on room temperature and relative humidity. Rapid desiccation is a concern since the smaller and lighter the infectious particle, the longer it will remain airborne [11,12].

Hence, even when infectious agents are expelled from the respiratory tract as large particles of mucus and saliva secretions, their rapid desiccation can lengthen the time they remain airborne [8].

Noteworthy is that both relative humidity and temperature—factors influencing the size/weight of the infectious aerosol and droplets—are strongly influenced by the production of water spray from dental hand-pieces and other DCU instruments.

## 5. Strategies and Solutions to Prevent Airborne and Aerosol Diffusion in Dental Clinics

WHO recommends the use of protective respirators as protective as N95 masks or FFP2 masks when performing aerosol generating procedures in most of medical branches [13]. In the present commentary the Authors propose new solutions to prevent the diffusion of aerosol from DCU and patient breathing in dental clinics.

Table 1, Table 2 and Table 3 resume some innovative concepts—new PPE for personnel, equipment and environmental recommendations—that must be considered in dental procedures to reduce the rebound of droplets containing microorganisms from the patients exhaled breath.

The main strategy that we suggest is the instant removal of the spray produced by hand-pieces and ultrasounds from the mouth-nose area, namely from the patient face and from the rubber dam surface (when present). We propose the use of some new designed suction devices positioned just around the mouth of the patient and on the side of the rubber dam or the patient lip.

A simple suction device may be a recently developed cheek retractor combined to a suction cannula to be positioned in the internal lip of patient to increase the saliva and droplet aspiration (lip suction cannula).

**Figure 2:**
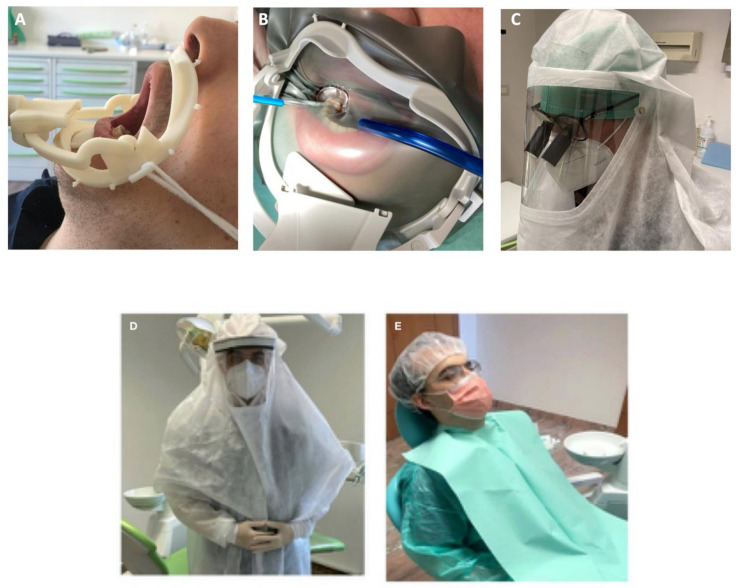
New solutions to prevent operator and patient’s contamination. (**A**) Preliminary version of resin 3D-printed device dental dam arch with a sliding suction cannula connected to the wider bore of DCU suction pump, designed during Covid-19 emergency to prevent aerosol diffusion of ultrasonic devices and burs. This system may be used for all aerosol-generating procedures, with or without dental dam sheet. (**B**) Commercial dental arch dam now in use in the Dental school to prevent water-saliva aerosol diffusion. The device differs from the prototype as it was designed with a wider operative area, having an easier access to the more distal teeth. It may be reused after heat sterilization cycles at 121 °C. (**C**) Individual waterproof sprayhood with polycarbonate transparent area which may be disinfected and cold sterilized. This device may be used in association with surgical loupes. Ventilation holes are present in the posterior area, in correspondence to the operator nape to avoid fog-up. (**D**) Operator equipment used during COVID-19 emergency and currently used at the moment in Bologna dental schools and dental clinics, constituted of a surgical sterilizable sprayhood, N95 respirators and sterilizable waterproof suits. (**E**) Patient vestment to avoid cross contamination during dental procedures.

Another new device is constituted by a double rubber-dam arch with a sliding suction pipe able to uptake spray produced in the mouth and by the nose. The authors contributed to the development of a preliminary 3D-printed version of the device reported in Figure 2A.

The recently introduced water saliva aerosol defender (WS Aerosol Defender, Cefla Medical Equipment, Modena, Italy), significantly reduced aereosol diffusion, according to the manufacturer declaration. A new commercial sliding suction pipe (Figure 2B) present a special configuration to be positioned just around the mouth in the more convenient position. The flow rate of 280–320 L/min has been declared when mounted on DCU wider suction bore. No clinical data are now available, however, the simultaneous use of conventional saliva ejector seems to not compromise the spray uptake. Further studies are being assessed.

The device can be applied with or without the rubber dam and potentially used in all oral procedures (i.e., surgery, extractions, hygiene procedures etc.).

When used in procedures that do not require dental dam application, the system may be easily applied and removed, in case of patients’ necessities, or operator procedures, such as dental impressions, or prosthetic rehabilitation check. Moreover, the suction pipe may be moved to different sites, in case of multiple rehabilitations.

**Figure 3:**
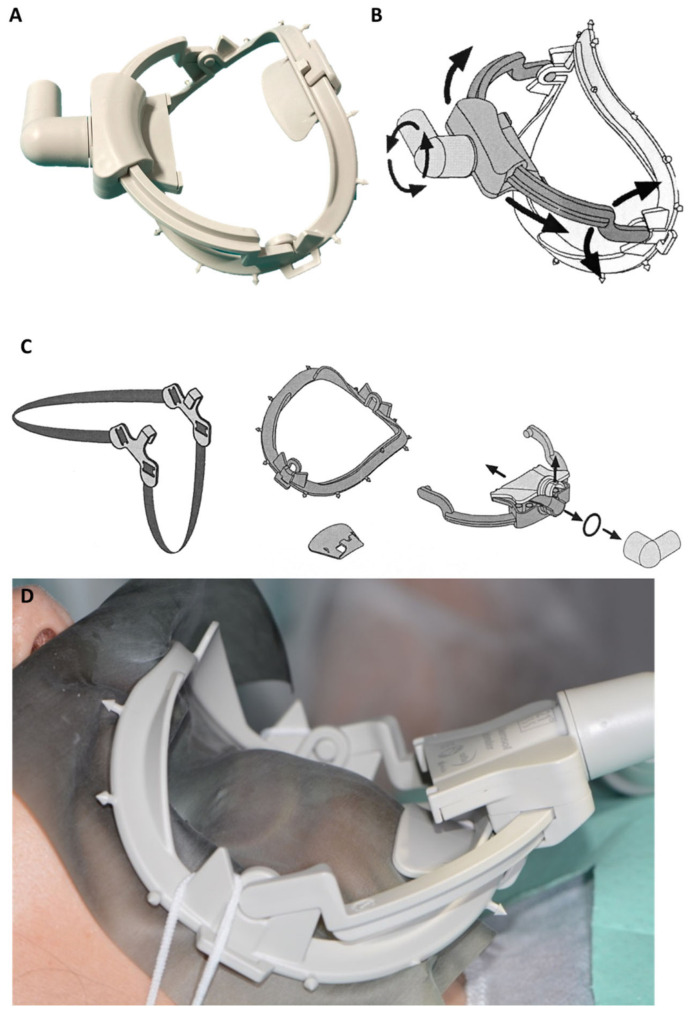
Images of Water saliva Air Defender, a new suction device designed to reduce aerosols dissemination during dental procedures. (**A**) The device is constituted of a dental dam arch connected to a sliding suction mouth. (**B**) The suction mouth may be moved according to the necessities on the lower or upper arch and on right or left quadrants. It is suitable for suction connections and complies with ISO 7494-2 “Dentistry Dental units Part 2: Air, water, suction and wastewater systems”. (**C**) Removal and disassembling of the device for autoclave sterilization procedures, which can be performed at 121 °C. (**D**) Schematic representation reporting the positionment on patient mouth.

The system is able to remove a large portion of spray produced in the oral cavity and may reduce the diffusion of spray and exhalation of patients. Information of this device use and its deployment is reported in Figure 3.

Innovative full-face impermeable sprayhood designed with a polycarbonate shield may ensure the complete closure of the face/neck region and the complete sealing of all the external surfaces that prevent spray penetration of contaminated aerosol. The use of sprayhoods is proposed as an additional physical barrier. Other studies demonstrated that the application of a physical barrier significantly reduce the aerosol diffusion [17,18,19].

Different models have been recently developed by many companies. Sprayhoods and similar devices such as surgical shield helmets are recently developed to prevent any contact of (infected) droplets with eyes and nose mucosa of operator and to avoid any particles inhalation (Figure 2C).

**Figure 4:**
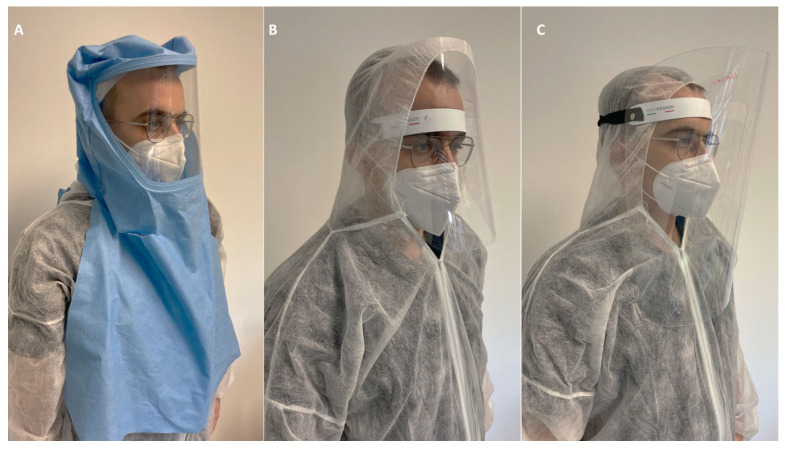
Three different sprayhoods tested during the Covid-19 emergency and used in post lock down period to prevent the operator contamination during dental procedures. All these devices may be cold sterilized and reused. (**A**) Full face impermeable sprayhood, lateral portion was reduced to prevent fog-up with breathing. (**B**) Protective cold sterilizable face mask which can be used in association with impermeable suit. (**C**) A modified version, with a larger polycarbonate shield to avoid droplets contamination from the lower margin of the shield, has been used in the dental clinic.

At this moment some different type of sprayhoods have been developed by different companies in Italy (Olisail, Trieste, Italy; Cartesio, Forlì, Italy; Cefla, Modena, Italy and by Biomet-Zimmer, Carlsbad, CA, USA) and have been introduced as routine device in ambulance staff and in Covid-19 hospital departments). Three different models, tested during the Covid-19 emergency and used in post-lock down period, are reported in Figure 4.

Sprayhoods must be used by DCWs during all the dental operative procedures and changed after each patient.

All the devices may undergo cold sterilization procedures and treatment with isopropyl alcohol and/or with 0.6% NaOCl solution to clean the entire external and internal surface. It is possible to use surgical loupes as visible in Figure 2C. To facilitate internal ventilation and to prevent fog up with breathing, some air holes are designed in the posterior area (in correspondence with the operator nape).

It may ensure a better fit and prevent large part of the inward leakage of aerosol derived from dental spray. A number of prototypes of sprayhood are being tested in dental schools, but no robust clinical data are available on their efficacy. However, in accordance with the maximum precaution principle the use of a sealing face shield may reduce the risk for spray contamination in operators such as dentists highly exposed for a long time.

A full-face piece powered air purifying respirator (PARP) connected with a blower and filter able to create positive pressure inside the face piece may be introduced in the dental clinics (especially for treatment of Covid-19 positive patients).

These samples have been tested in general surgery and otorhinolaryngologic surgeries hospitals. Their efficacy must be proved in future especially considering that new developed facemasks are now produced for critical patients.

Tyvek suits must be used by all the dental operators and usually covered by monouse gown, spray hoods and obviously facemask. All university dental schools in Italy are now working with these devices. All large rooms of many university dental schools in Italy have been reorganized. In Bologna University we have introduced Plexiglas and drywall divisions to isolate any dental chairs (with the creation of boxes completely closed by doors and isolated).

All daily organization have been redesigned to prevent virus diffusion caused by fluctuating droplets [4]. Dentists must immediately change their operative procedures and try to reduce spray droplets and particles production. They have to innovatively improve all environmental disinfection procedures for rooms and laboratories surfaces (Table 3). Reception personnel must avoid any contact with the surgery rooms where spray droplets may be captured by clothes and hairs if not protected.

## 6. Salivary Diagnostics to Detect SARS-CoV2 Positive Patients and Their Potential Use in Dental Clinics

After lockdown period, the attention moved to the detection of the high number of Sars-CoV2 asymptomatic patients. Real time PCR of nasopharyngeal swabs are currently standard technique to detect Sars-CoV2 positive patients [20].

However, some drawbacks are reported, including the long time to achieve a response (24–72 h) invasiveness for the patients and potential risk of transmission of the operators [21].

Recently, a lateral flow immunochromatographic assay was designed (Biocredits, BioVendor, Brno, Czech Republic), to detect Sars CoV-2 antigens, which may be potentially used in dental clinics and university departments due to the reduced processing time (approx. 5 min). The test showed comparable sensitivity and specificity percentages were reported when used in collecting nasopharyngeal swabs in patients with high viral load samples (>80%). The possibility to be used also in other oral fluids, such as sputum or saliva has been investigated, showing however lower capability of detecting Sars-CoV2, the values reported were 28.6% and 56.85% [22].

Other tests are being implemented in clinical practice, to overcome these limitations.

Saliva is a potential source of Sars-Cov2 contamination, the presence of Sars-CoV2 in saliva is related to different access ways, namely the direct access of the virus into the oral cavity via lower/upper respiratory tract, through gingival crevicular fluids or via salivary fluids from infected salivary glands [21,23]. Due to the high presence of Sars-Cov2 replicas, saliva recently gained particular attention as a potential new approach for COVID-19 diagnosis [24,25].

Salivary diagnostics shows numerous advantages when compared to traditional nasopharyngeal swabs: it is low invasive, relatively cheap and easier to be handled and collected, possibly avoiding operator contamination [21]. Recent studies revealed a comparable detection rate of Sars-CoV2 in saliva, when compared to nasopharyngeal swabs, the range was 85–91% according with several studies [24,26,27].

The two main strategies of salivary tests are detection of Sars-CoV2 spike protein [24,28,29] or anti-SARS-CoV-2 IgG and IgM [30]. Innovatively new salivary tests allow the detection of in saliva in less than 5 min with a 90–95% sensitivity [24,28,29].

IgM and IgG test cassettes detection kit is based on a chromatographic lateral flow immunoassay for the qualitative detection in 15 min of anti-SARS-CoV-2 IgG and IgM in human whole blood, serum or plasma samples [30].

Implementation of these new fast tests may play a positive role in dental clinics and dental schools. Dentists may be able to perform saliva screening or IgM IgG rapid detection tests to intercept symptomless and low symptomatic patients with virus infection.

## 7. Conclusions

In conclusion, the great number of patients needing routine dental treatments in the SARS-CoV-2 s pandemic wave still requires great attention. The risk may result extremely high by the presence of asymptomatic patients positive for coronavirus. An apparently healthy patient may potentially create the condition for a sequence of serious contaminations. For these reasons, HDWs i.e., dentists, dental staff personnel, students and patients must be protected (Figure 2D,E), in particular whose affected by systemic diseases [31].

The present commentary wants to emphasize the need for novel protection measurements from droplets spray for dental staff and patient and new operative approaches in the management and organization of the dental service.

In all cases the new dental routine proposed should be maintained for a long time, even after the development of a new SARS-CoV2 vaccine. New cases of seroconversion and secondary infection of COVID-19 recovered patients are reported in literature, and there are few data on the duration of immunity after Sars-CoV2 infection [32]. The Covid-19 era is imposing a new pragmatic approach to future dental routines.

## Figures and Tables

**Table 1:** Proposed equipment to reduce by removing spray diffusion produced by the water-cooled handpiece and by the nasal breathing of the patient.

1	Oral lip aspiration kit to be applied into the internal lip area for spray suction;
2	New aspiration kit with multiple aspiration cannulas connected with a new adjunctive (powerful) pump suction to adapt to pre-existent DCU or to use in association with;
3	Novel designed kit constituted by a composite dental dam arch with a sliding suction cannula (Water-saliva-aerosol (WSA) defender system) connected with DCU suction pump. The system may be applied around the patient’s mouth as a normal dam arch (with/without the rubber sheet);
4	Use of oral disinfectant solutions such as povidone-iodine 0.23%, [14] hydrogen peroxide [15] in association with silver particles [16], as mouth-washing/antimicrobial rinses (before any clinical procedures) to reduce the viral load of saliva and droplets generated by spray eventually contaminated by patient;

**Table 2:** Proposed solutions to prevent personnel contamination. All these equipment and procedures should be used in association for all patients to try to reduce the contamination risks.

1	Use of N95 (FFP2/3) respirators for all patients and all PPE such as gloves, glasses, gown etc.;
2	-Individual waterproof sprayhood as protective hat (helmets with cover) with a solid sealing transparent area for all dental staff, as special PPE to use face-to-face with the patient may help to prevent spray droplet contact with neck, eyes, face; or alternatively: -Full facepiece Powered Air Purifying Respirators (PARPs) with blowers to create positive pressure inside the facepiece for example in emergency with Covid-19 positive patients;
3	Tyvek suit full body protection; a mono-use gown may be applied on Tyvek suits. Alternatively use of impervious disposable gown with head cap;
4	Waterproof protection for shoes and for trousers to avoid the collection of wet and dry contaminated droplets from office floor that must be used with gowns and other devices;

**Table 3:** Proposed solutions and new processes to prevent environment contamination in dental clinics surfaces.

1	Removal of any small objects and boxes from DCU and technical furnishing (i.e., cotton roll containers, drills, bur boxes, endodontic instruments, composite resin tubes, etc.). Use of 70% isopropyl alcohol and to disinfect any small objects and devices manipulated during the clinical procedures (i.e., composite tubes, light-curing units etc.) [15]. All in-office surfaces furniture must be flush-deck and easy to clean;
2	All suction circuit pipes and DCU sink must be irrigated with disinfectants such as 0.5% sodium hypochlorite solutions immediately after patient discharge to remove and to break down any infected reflux from hydraulic circuits;
3	Use 0.1% sodium hypochlorite and 70% isopropyl alcohol solutions to clean and remove any droplets deposited on surfaces and objects present in the room and exposed to droplets contaminations (i.e., radiographic device; operator chair; endodontic microscope etc.);
4	Removal of any biological wastes from each patient (i.e., gowns, towels etc.) and rapidly sealed up and isolated inside plastic containers or trash bags to avoid secondary aerosolization. After all cleaning procedures for DCU and dental clinics (approximately 15–30 min) all operators must remove their exhaust gown, mask, sprayhood and inserted in another plastic trash bags before replacing them with new disposables/gown etc. for the next patient.
